# The Role of Diet as a Modulator of the Inflammatory Process in the Neurological Diseases

**DOI:** 10.3390/nu15061436

**Published:** 2023-03-16

**Authors:** Antonina Kurowska, Wojciech Ziemichód, Mariola Herbet, Iwona Piątkowska-Chmiel

**Affiliations:** Chair and Department of Toxicology, Faculty of Pharmacy, Medical University of Lublin, Jaczewskiego 8b Street, 20-090 Lublin, Poland

**Keywords:** inflammation, anti-inflammatory diet, nutrition intervention, neurological diseases, neurodegenerative diseases, mental disorders

## Abstract

Neurological diseases are recognized as major causes of disability and mortality worldwide. Due to the dynamic progress of diseases such as Alzheimer’s disease (AD), Parkinson’s Disease (PD), Schizophrenia, Depression, and Multiple Sclerosis (MD), scientists are mobilized to look for new and more effective methods of interventions. A growing body of evidence suggests that inflammatory processes and an imbalance in the composition and function of the gut microbiome, which play a critical role in the pathogenesis of various neurological diseases and dietary interventions, such as the Mediterranean diet the DASH diet, or the ketogenic diet can have beneficial effects on their course. The aim of this review was to take a closer look at the role of diet and its ingredients in modulating inflammation associated with the development and/or progression of central nervous system diseases. Presented data shows that consuming a diet abundant in fruits, vegetables, nuts, herbs, spices, and legumes that are sources of anti-inflammatory elements such as omega-3 fatty acids, polyphenols, vitamins, essential minerals, and probiotics while avoiding foods that promote inflammation, create a positive brain environment and is associated with a reduced risk of neurological diseases. Personalized nutritional interventions may constitute a non-invasive and effective strategy in combating neurological disorders.

## 1. Introduction

The nervous system includes the brain and spinal cord, which together are the central nervous system (CNS). The brain integrates the received information and coordinates and influences the activity of all parts of the body [1]. The functioning of the CNS is dependent on many factors both internal and external. Internal factors, called neurotrophic elements, influence the CNS by controlling nerve tissue’s survival, growth, and operation. They affect the target cells by activating numerous signaling pathways and distinct kinds of receptors. Nerve growth factor (NGF) alongside brain-derived neurotrophic factor (BDNF), with neurotrophin-3 (NT-3) and neurotrophin-4 (NT-4) are examples of “classic” neurotrophins. The functioning of the brain also depends on various external factors listed below.

Stress, an unhealthy lifestyle, and an unbalanced work-to-rest schedule alongside the progression of environmental pollution cause mental and neurological diseases that manifest in patients at all ages. Both the WHO (World Health Organization) and the European Brain Council (EBC) present alarming data concerning the morbidity of Central Nervous System diseases. According to WHO data, by the year 2030, unipolar depression will overtake all other occurring major oncological diseases and metabolic disorders. The ECB estimates that one in three Europeans suffers from a brain disorder at least once a year [2,3]. According to research by the European Brain Council, there are roughly 15 million people in Poland who have neurological problems. While the number of illnesses brought on by infections is declining, cerebrovascular disorders and neurodegenerative diseases are on the rise in the Central European region [2].

Neurological diseases are often associated with aging of the society. Currently, in youngsters, the most common conditions being treated are meningitis and epilepsy, whereas neurodegenerative diseases, including Alzheimer’s or Parkinson’s, occur mostly among the elderly. Disability-adjusted life year rankings (DALY) made for Eastern Europe indicate that strokes and migraines rank first and second in terms of incidence. Alzheimer’s disease placed third, followed by brain and nervous system tumors, drug-related headaches, epilepsy, other neurological disorders, multiple sclerosis, Parkinson’s disease, tension headache, meningitis, encephalitis, motor neuron disease, and tetanus [2,3,4]. Diseases of the central nervous system are serious health problems that can lead to death or disability [5]. The process of how neurological illnesses develop is highly complex as there are many potential causes. The particular causes of neurological issues may include brain injury, spinal cord injury, or nerve injury. Genetic and environmental factors, congenital anomalies, infections, and unhealthy lifestyles also belong among the potential reasons for central nervous system diseases. Several neurological conditions have gender differences in prevalence or outcome. For example, autism is more common in boys, depression in women, Parkinson’s disease in men, and multiple sclerosis in women. In the case of a stroke, women have a less favorable outcome and experience a more precipitous decline in health status than men [6].

Free radicals, which are largely produced by molecular oxygen, are thought to increase the risk of a number of health problems, including aging and neurological diseases [7]. Free radical damage to tissue biomolecules, such as lipids, proteins, and DNA, is hypothesized to play a significant role in the pathophysiology of oxidative stress. At the cellular level, one of the main causes of disorders of the central nervous system is oxidative stress [7]. The etiology of neurodegenerative disorders, which includes Alzheimer’s disease, amyotrophic lateral sclerosis, Huntington’s disease, Parkinson’s disease, or stroke (brain ischemia/reperfusion injury) is intimately linked to oxidative damage [7].

According to the findings of current studies, dietary choices have an impact on molecular processes that control energy metabolism and synaptic plasticity [8,9,10,11]. The influence of nutrients on factors consisting of microbiome composition, microbial metabolites, gastrointestinal signaling molecules, and neurotransmitters strongly indicates that dietary patterns can affect the development of metabolic changes and inflammation [8,9]. Research has suggested that alterations in the composition and function of the microbiota may be associated with the development and progression of a range of psychiatric disorders, including depression, anxiety, and schizophrenia. The microbiota is known to influence the functioning of the gut-brain axis through the production of metabolites, such as short-chain fatty acids, that can act on the central nervous system. In addition, alterations in the gut microbiota have been associated with changes in the levels of neurotransmitters, such as serotonin and dopamine, which are involved in the regulation of mood and behavior. Some studies have suggested that probiotics may have beneficial effects on depression and anxiety symptoms, and cognitive function [12,13,14].

The hypothesis that nutrients can be ingested through whole foods and dietary supplements to lessen the effects of brain damage is supported by research results in animal models of central nervous system impairment. A noninvasive and practical method to assist in *n* the treatment of neurologic and cognitive diseases appears to be diet and exercise [5]. In recent years, the emerging role of nutraceuticals containing vitamins, minerals, amino acids, fatty acids, or plant extracts in supplement form was highlighted [15]. The ideal quantity of sleep, hydration, and proper diet is crucial for the effectiveness of our minds [7]. Unfortunately, ultra-processed food consumption has been rising all across the world. People are eating more ultra-processed foods that are high in saturated fats and carbohydrates, which in the long haul have a negative impact on the human body, especially on the brain [8]. In order to increase the shelf life, improve the appeal, and increase profitability, ultra-processed foods contain little to no naturally occurring ingredients [9]. Preservatives, stabilizers, emulsifiers, solvents, and binders alongside bulking agents, sweeteners, sensory enhancers, flavorings, and colorings make up the majority of an ultra-processed food’s constituents [10]. Nutritionally speaking, these are not foods that should be consumed frequently and in large quantities [10]. Artificial preservatives, which include those synthetic or semi-synthetic compounds, such as benzoates, sorbates, nitrites, and nitrates of potassium or sodium, as well as potassium sulfites, glutamates, and glycerides, are mostly considered safe, but several have negative effects for the consumer health [10]. It is challenging to predict the long-term effects of a single component found in small concentrations in food products on a system as complicated as the human body [9,10]. Though, there is evidence that excessive consumption of the above-mentioned compounds might be unhealthy and can hasten the onset of chronic diseases [8,9,10].

Simple sugars as well as saturated fatty acids are proven to be related to memory problems and hippocampus damage [16]. Studies on mice showed that exposure to “high-fat diets” (40–65% of daily calories coming from fat) or simple sugars (sucrose or high fructose corn syrup) from infancy can adversely impact hippocampus-dependent learning and memory functions [16,17]. These deficiencies can sometimes continue throughout adulthood despite nutritional intervention and happen independently of obesity and metabolic disturbance [16]. According to data from the World Health Organization, malnutrition is responsible for 45% of infant deaths worldwide, which occurs mainly in less developed countries. On the other hand, in the Western world, excessive caloric intake, and a lack of physical activity have become big problems in recent years, which can lead to weight gain and associated health issues. To promote good health, it is recommended to maintain a balanced and varied diet that includes plenty of fruits, vegetables, whole grains, lean proteins, and healthy fats with regular physical activity. The brain needs a lot of energy to properly function. It uses glucose as a basic fuel to process data and store it in memory. However, a diet excessive in simple carbohydrates is proven to have a negative impact on patients’ brain vessels [17]. Recent studies reveal that an ultra-processed diet’s high intake of simple carbohydrates and saturated fats alter the redox state, the gut microbiota, and the inflammatory response [18,19]. The brain integrates the received information and coordinates and influences the activity of all parts of the body [1]. The functioning of the CNS is dependent on many factors both internal and external. Internal factors, called neurotrophic elements, influence the CNS by controlling nerve tissue’s survival, growth, and operation. They affect the target cells by activating numerous signaling pathways and distinct kinds of receptors. Nerve growth factor (NGF), brain-derived neurotrophic factor (BDNF), neurotrophin-3 (NT-3), and neurotrophin-4 (NT-4) are examples of “classic” neurotrophins. The functioning of the brain also depends on various external factors listed below.

The objective of this review is to examine in depth the influence of diet, including its inflammatory properties, on the onset and/or advancement of central nervous system disorders.

## 2. Mechanism of Action Associated with Diets and Neurological Disease Development

### 2.1. Diet, Inflammation, and Its Impact on Neurological Disease Development

Diet and inflammation are interconnected and play a crucial role in the development of neurological diseases such as Alzheimer’s disease, Parkinson’s disease, multiple sclerosis, schizophrenia, bipolar disease, and depression. Dietary factors have been identified as key modulators of inflammation. A diet rich in whole foods, including fruits, vegetables, whole grains, and lean protein sources, has been associated with lower levels of inflammatory markers, such as C-reactive protein (CRP) and interleukin-6 (IL-6). Conversely, a diet high in processed and high-fat foods, refined carbohydrates, and red meat has been linked to increased inflammation and a higher risk of chronic diseases, including neurological disorders.

#### 2.1.1. Inflammation-Promoting Diet

An inflammation-promoting diet is a diet that contains foods that can trigger an inflammatory response in the body. Over the past few decades, there has been a notable shift in the dietary habits of Westernized countries toward increased consumption of Western-type diets (WDs). These diets are characterized by a high intake of processed meals, convenience foods, snacks, sugary soft drinks, other high-fat foods, and carbohydrates. The majority of constituents in an ultra-processed food consist of preservatives, stabilizers, emulsifiers, bulking agents, solvents, binders, sensory enhancers, sweeteners, flavorings, and colorings. Nutritionally speaking, these are not foods that should be consumed frequently and in large quantities.

High levels of saturated and trans fats are found in animal products, such as meat and dairy, and in processed foods, such as chips, cookies, and fried foods. These fats increase inflammation by promoting the production of pro-inflammatory cytokines, such as IL-1, IL-6, and TNF-alpha [13,14]. According to research, a diet high in SFAs (saturated fatty acids) and TFAs (trans fatty acids) has also been linked to an increased risk of Alzheimer’s disease [20]. Studies have indicated a strong association between increased intake of total fats, SFAs, and increased risk of cardiovascular disease, and decreased cognitive function [21]. On the other hand, healthy adults who consumed a diet low in fat but high in omega-3 fatty acids experienced protection from cognitive deterioration [20,21].

Furthermore, the consumption of polyunsaturated fats (PUFAs), particularly *n*-3 PUFAs, has been linked to a reduced risk of cardiovascular mortality due to their anti-inflammatory properties. Some studies suggest that diets high in monounsaturated fatty acids (MUFA) may also have anti-inflammatory effects [17]. The balance between *n*-6 and *n*-3 fatty acids is of great significance. The amount and type of fat consumed significantly impact the degree of inflammation and the likelihood of developing non-communicable diseases [22,23,24,25,26].

Another component of an inflammation-promoting diet is a high intake of refined carbohydrates, such as white bread, pasta, and sugary drinks. These foods cause a rapid increase in blood sugar levels, leading to the release of insulin [16]. Prolonged high insulin levels can contribute to the development of insulin resistance in tissues, which is one key factor in the pathogenesis of inflammation. According to studies, consuming high GI foods frequently is positively correlated with having higher serum levels of inflammatory markers such as CRP, TNF-α, and IL-1β, IL-6 [18,19]. Whereas a large intake of dietary fiber has the opposite effect [27].

Moreover, an inflammation-promoting diet is low in antioxidant-rich fruits and vegetables, which can neutralize free radicals and decrease inflammation. A diet lacking in fruits and vegetables has been associated with increased levels of oxidative stress and inflammation markers that negatively affect our physical and mental health [17].

##### Dietary Inflammation Index (DII) 

The impact of diet on inflammation has been recognized, and in 2004, the Dietary Inflammation Index (DII) was developed to evaluate the potential effect of a diet on inflammation status. The DII categorizes diets on a spectrum from highly anti-inflammatory to highly pro-inflammatory and is calculated using 45 food components and nutrients to determine the diet’s potential impact on inflammation. Because of the extensive research foundation and added improvements, the DII is widely applicable [28,29,30,31].

A more pro-inflammatory diet has a higher DII score, whereas an anti-inflammatory diet has a lower DII score [32]. The DII score is typically derived from validated FFQs or previous dietary records in practical applications [33]. The DII score has a substantial correlation with the risk of illness, for example, cardiovascular diseases [21].

#### 2.1.2. The Pyramid of Anti-Inflammatory Nutrition

The anti-inflammatory diet pyramid was created using the anti-inflammatory food components mentioned in later sections of the manuscript as a foundation. There are two ideas for such pyramids that we have found so far in scholarly literature. In a study from 2012, Weil proposed an approach including 16 different food groups. Water, vegetables, fruit, legume seeds, whole grains, pasta, healthy fats (nuts and seeds, olive oil, avocado), fish and shellfish, soy products, Asian mushrooms, and additional protein sources (dairy, eggs, poultry, lean meats), spices and herbs, teas, dietary supplements, red wine, and healthy sweets like dark chocolate are the products that we should consume the most [34].

In 2018, a group of researchers developed a new pyramid with a slightly different division. During the first visit, the patient’s food consumption was evaluated using the Block Brief 2000 Food Frequency Questionnaire (FFQ), a method that has been scientifically verified [35].

The construct validity of the DII^®^ was evaluated in a population using multiple 24-h dietary recall interviews and up to five 7-day dietary recalls. The evaluation and scoring concerned 1943 publications. Six specific inflammatory markers, including CRP, IL-1, IL-4, IL-6, IL-10, and tumor necrosis factor (TNF-α), were used to identify 45 dietary parameters, including foods, minerals, and other bioactive components. Each of the 45 factors was compared against a global database that is geographically representative and includes diet surveys from 11 different nations (i.e., nutrients, and other food components). The DII^®^ scores were determined using intake information taken from this database [35].

Studies have demonstrated that an anti-inflammatory diet can be a beneficial nutritional approach in the management of obesity, cardiometabolic, and autoimmune disorders (including inflammatory bowel diseases as well as rheumatoid arthritis), cancer (including breast cancer), neurodegenerative diseases (Alzheimer’s disease), as well as depression [36,37]. The eating patterns with previously documented health benefits, such as the Mediterranean, DASH, and vegetarian diets, meet the majority of the assumptions of the anti-inflammatory diet. Table 1 outlines that these diets endorse the intake of abundant fruits and vegetables, wholewheat products, healthy fats, and sources of protein derived from either plants or animals [38,39].

#### 2.1.3. Antiinflammatory Components of the Diet

The influence of consuming foods on human neurologic function has been an interest of researchers for decades. An increasing body of evidence suggests that certain components of the diet have a beneficial impact on the onset and advancement of different neurological disorders [8,49]. Below, we present the published information on dietary components with high anti-inflammatory and antioxidant activity.

##### Fruits and Vegetables

Research indicates that consuming a diet rich in fruits and vegetables is associated with lower concentrations of inflammatory indicators. Consuming fruit and vegetables, which are abundant in bioactive substances including polyphenols and antioxidant vitamins, minerals, and fiber, is inversely correlated with inflammation and oxidative stress. The available research shows that adult consumption of fruits and vegetables is negatively correlated with pro-inflammatory cytokines and reactive oxygen species, which are related to inflammation and oxidative stress. To lower pro-inflammatory cytokines and reactive oxygen species, it is recommended to consume five portions of fruits and vegetables on a daily basis [35]. Various narrative reviews and cross-sectional studies indicate that consuming a diet abundant in fruits and vegetables is associated with reduced concentrations of inflammatory markers and oxidative stress (such as urinary 8-iso PG F2α, an F2-isoprostane) [50,51]. Saita et al.’s review of epidemiological studies revealed that plant products can have positive effects on the cardiovascular system by limiting LDL cholesterol oxidation, thereby slowing down atherosclerotic plaque formation [49].

##### Herbs and Spices

Due to their high biological activity, herbs, and spices are a great complement to a variety of cuisines because of their flavor and odor qualities as well as their positive impacts on health, as noted in literature reviews [52,53].

The antioxidant and anti-inflammatory effects of herbs and spices have been attributed to three primary categories of compounds: terpenes, phenolic acids, and flavonoids. They could either enhance the activity of anti-inflammatory enzymes, factors, and proteins or disrupt these pathways in the inflammatory process [54].

Curcumin and garlic appear to offer the most potent anti-inflammatory effects by reducing the concentrations of inflammation biomarkers in the bloodstream. Turmeric is a spice that contains curcumin, a polyphenolic compound. It has been used for centuries to manage inflammatory conditions and is utilized both on its own and as a component of spice blends, such as curry powder [54]. Numerous studies conducted over the last 20 years have indicated that curcumin can exert anti-inflammatory effects through its regulation of various biological targets. These effects involve the suppression of inflammatory transcription factors, enzymes (such as COX-2 and LOX-5), and cytokines [54].

Garlic, *Allium sativum L.,* has been used for both culinary and medicinal purposes for a long time due to its distinctive aroma and taste. According to several reports, including garlic in your diet may lower your chance of developing cardiovascular diseases [55]. Furthermore, garlic has been demonstrated to possess various beneficial properties, including anti-inflammatory, antioxidant, anti-hypercholesterolemic, and antithrombotic effects. Bioactive compounds with a strong anti-inflammatory effect are allicin, diallyl sulfide, diallyl disulfide, diallyl trisulfide, quercetin, and kaempferol (Table 2). According to research, garlic extract may be a potential treatment for inflammatory bowel disease due to its ability to suppress the production of inflammatory cytokines including Tumor Necrosis Factor-α, interleukin 1β, 6, and interferon-C [55].

##### Zingiber Officinale

Another important spice, ginger (*Zingiber officinale* Roscoe), has been used extensively in ethnomedicine for many years. Zingiber officinale contains a variety of components including lipids (such as free fatty acids), amino acids, oleoresin-bearing phenolic compounds, carbohydrates (of which starch is a prominent component), lipids, and a volatile oil containing a mixture of terpenes and phenolic chemicals. Terpenes and phenolic compounds, particularly gingerols, are the most common physiologically active substances. It is known that ginger formulations have anti-inflammatory, antioxidant, and analgesic properties [56]. When given to rats orally or intravenously, high doses of ginger (500 mg/kg) were significantly effective in lowering serum PGE (2). TXB (2) levels were significantly lower in rats given 500 mg/kg ginger orally but not intraperitoneally. A higher dose of ginger (500 mg/kg) resulted in a significant reduction in serum cholesterol. Only when ginger was administered IP did a significant reduction in serum cholesterol be observed at a low dose of ginger (50 mg/kg). There were no significant changes in serum triglyceride levels after either the low or high dose of ginger was administered. These findings imply that ginger may be useful as a cholesterol-lowering, antithrombotic, and anti-inflammatory agent [56] (Table 2). Initially, it was believed that the anti-inflammatory effects of ginger were due to the simultaneous inhibition of the key enzymes involved in the metabolism of arachidonates, namely cyclooxygenase (COX) and 5-lipoxygenase (LOX), but further research has revealed that it also involves the downregulation of the expression of pro-inflammatory genes [56].

Recent studies suggest that ginger preparations may have a positive impact on COVID-19-related lung inflammation, due to their anti-inflammatory and immunomodulatory properties [57,58].

*Rosmarinus officinalis* L., a herb native to the Mediterranean region, is renowned for producing an antimicrobial essential oil and is now found worldwide. Traditional medicine has recognized the antioxidant and anti-inflammatory properties of its leaf extracts and decoctions, which are used to treat skin conditions like eczema. The crude extract of *R. officinalis* L. and its derived fractions, along with isolated compounds, have demonstrated significant anti-inflammatory effects. These effects are believed to be due to the presence of carnosol, betulinic acid, and ursolic acid, which inhibit the release of pro-inflammatory mediators like NOx, IL-1, and TNF-1α while reducing leukocyte activation at the site of inflammation [59,60,61].

**Table 2:** Herbs and spices as a source of compounds with anti-inflammatory potential.

Herbs and Spices	Major Bioactive Compounds	Potential Beneficial Effects	Anti-Inflammatory Mechanism	References
Garlic (*Allium sativum* L.)	allicin, quercetin, kaempferol	anti-inflammatory	suppress the production of inflammatory cytokines	[55]
Turmeric (*Curcuma longa* L.)	curcumin, demethoxycurcumin, bisdemethoxycurcumin	anti-inflammatory	inhibits microglial activation and reduces cytokine release	[54]
Ginger *(Zingiber officinale* Roscoe)	gingerols, shogaols, paradols	anti-inflammatory antioxidant analgesic	Inhibit cyclooxygenase (COX) and 5- lipoxygenase (LOX), and downregulation of the expression of pro- inflammatory genes suppress the production of inflammatory cytokines	[56,57,58]
Rosemary (*Rosmarinus officinalis* L.)	carnosic acid, betulinic acid, carnosol, ursolic acid	anti-inflammatory antioxidant	inhibit the release of pro-inflammatory mediators like NOx, IL-1, and TNF-1α while reducing leukocyte activation	[59,60,61]

Fatty salt-water fish and other *n*-3 sources.

Oily marine fish is the most abundant and easily accessible source of *n*-3 fatty acids. They contain abundant amounts of long-chain fatty acids, specifically EPA and DHA. It has been demonstrated that they possess anti-inflammatory action in addition to anti-arrhythmic, anti-hypertensive, and anti-aggregating capabilities. They also lower blood triglyceride levels, making them an effective cardiovascular incident risk reduction [49]. Another source of *n*-3 acids is seaweed. Also, α-linolenic acid, a precursor to EPA that has a lipomizing effect, can be obtained from the diet. Linseed, linseed oil, walnuts, and to a lesser extent, rapeseed oil are some of its sources [49].

##### Vegetable Protein: Soybeans and Other Legumes

Legume seeds include a variety of nutrients, including fiber. Additionally, they are a strong source of vegetable protein. Soy isoflavones (ISFs) have been found to possess anti-atherosclerotic, antioxidant, antiproliferative, and anti-amyloidogenic properties in preclinical studies [62]. Additionally, several studies have shown that soy products have health benefits in preventing various diseases such as heart disease, obesity, cancer, diabetes, and osteoporosis, as well as regulating blood pressure [63].

Therefore, it is advised to substitute some vegetarian protein for animal protein in patients with chronic diseases associated with inflammation [49,64]. Moreover, it has been indicated in recent studies that equally, a metabolite generated in the gut after consuming soy products, may lower the risk of developing dementia [65].

##### Vegetable Oils Rich in Unsaturated Fatty Acids

Olive oil is an example of a healthy fat with various health benefits. Olives and olive oil are abundant in monounsaturated fatty acids that can modify anti-inflammatory pathways and gene expression, leading to a reduction in inflammation. It has a favorable linoleic acid/alpha-linolenic acid ratio; it is high in monounsaturated fats and low in polyunsaturated fats. It also includes phytosterols, polyphenols, vitamin E, vitamin A, and phytosterols [66]. Its favorable composition, and high levels of oleic acid, vitamin E, and polyphenols, make it resistant to oxidative changes. Phytosterols work together to minimize cholesterol absorption. These antioxidant properties can play a crucial role in preventing or ameliorating various diseases that are associated with oxidative stress, such as atherosclerosis and other chronic degenerative disorders [24,66].

Growing evidence indicates that consuming extra virgin olive oil (EVOO) regularly is associated with a reduced risk of chronic degenerative diseases like cardiovascular disease, type 2 diabetes, and cancer [67]. The health benefits of EVOO are attributed not only to its monounsaturated fat content but also to the presence of phenolic compounds, which have antioxidant, anti-inflammatory, and immunomodulatory properties [49,67]. In vitro and in vivo studies demonstrate that EVOO and its polyphenols can alleviate disease symptoms in IMID patients by acting at the local and systemic levels, modulating multiple molecular pathways [68,69].

Another anti-inflammatory fatty acid, gamma-linolenic acid, a member of the *n*-6 family, can be added to the diet to treat disorders with severe inflammation. Evening primrose oil, borage oil, or blackcurrant seed oil are alternative sources of this compound [70].

##### Nuts and Seeds

Incorporating nuts and seeds as snacks or in meals can support the anti-inflammatory effects of the diet, as they contain nutrients such as dietary fiber, phytonutrients, vitamins, minerals, and essential fatty acids that aid the body in healing from inflammation. First of all, high consumption of pumpkin seeds, pistachios, walnuts, and almonds is advised due to their high content of antioxidants [35]. Nuts contain active compounds that have the potential to enhance the body’s endogenous antioxidant defense and regulate the cellular redox state. A review from 2018 gathered information on the positive impact of three nuts: almonds, hazelnuts, and walnut on patients with Alzheimer’s disease [71].

Several studies have indicated that nuts contain micronutrients and phytochemicals that can impact various pathways involved in Alzheimer’s disease, including amyloidogenesis, tau phosphorylation, oxidative stress, and cholinergic pathways [71,72].

##### Tea Beverages

Widespread traditional usage of tea—both black and green—contributes significantly to the diet’s capacity for reducing inflammation. It has been demonstrated that green tea extract, which is high in phenolic compounds, has an antioxidant capacity and inhibits LDL-cholesterol oxidation. Due to its anti-inflammatory and antioxidant characteristics, tea can serve as a partial substitute for water in quenching thirst while lowering the progress of disease [53].

##### Coffee

Research has suggested that caffeine, a stimulant found in coffee, tea, and chocolate, may have a protective effect against Alzheimer’s disease, a progressive brain disorder. While the exact mechanism by which caffeine may protect against Alzheimer’s disease is still being studied, it is thought that caffeine may help to reduce inflammation and protect brain cells from damage. Studies showed that caffeine intake is associated with a lower risk of developing Alzheimer’s disease in older adults than those who do not drink coffee [72,73,74]. However, more research is needed to fully understand the relationship between caffeine and Alzheimer’s disease.

##### Red Wine

Increased research interest has been sparked by the link between dementia and alcohol use/abuse. With the growing consumption of wine worldwide, several studies have been conducted to examine if it could be a modifiable risk factor for cognitive impairment. Direct neurotoxic effects increase the risk of dementia through excessive wine consumption; however, light to moderate wine consumption appears to reduce the risk of dementia and cognitive decline in an age-dependent manner [75]. The presence of polyphenols, especially in red wine, and their antioxidant properties contribute to these beneficial effects of moderate consumption. Resveratrol (3,5,4′-trihydroxystilbene), a polyphenolic compound found in grape and red wine belonging to the stilbene family, is responsible for this positive effect, according to research. Regular low-dose resveratrol supplementation can enhance cognition alongside cerebrovascular function [75,76]. Due to the high concentration of bioactive substances (polyphenols) and their anti-inflammatory effects, red dry wine can be consumed in moderation (no more than 1 glass per day). However, it is not additionally advised for the prevention of cardiovascular disorders in nondrinkers [35].

##### Dark Chocolate

Cocoa, which is the main ingredient in chocolate, is abundant in biologically active compounds that possess anti-inflammatory and antioxidant properties. The results of recent studies show the positive impact of cocoa flavanols on cognitive function as well as neuroplasticity [35]. There is a mounting body of evidence indicating that cocoa and cocoa products have a positive impact on human cognition, particularly in elderly populations and patients at risk [35,53]. The favorable effects are associated with an increase in cerebral blood flow or oxygenation of the brain following acute consumption. In addition, regular intake of cocoa flavanols has been linked to enhanced cognitive performance and elevated levels of neurotrophins in young adults [53].

### 2.2. Diet and Microbiome and Its Effects on Neurological Disease Development and Its Outcomes

Amounts as well as the composition of the diet play a significant impact in determining the structure and function of the human microbiota. Recent research has shown that the gut microbiome can influence the development and outcomes of neurological diseases [17,77]. A healthy gut microbiome is essential for sustaining a healthy organism, by positively affecting metabolic and immunological functions [78]. Due to the presence of cells resembling those found in the brain, the gut is frequently referred to as “the second brain.” The microbiota that inhabits the gut play a critical role in regulating overall brain function through a variety of pathways, including immune, endocrine, and vagal routes, influencing hormones, neurotransmitters, cytokines, and short-chain fatty acids [79,80].

Preclinical investigations suggest that administering probiotics can lessen both peripheral and central inflammation by reducing the levels of IL-6 and TNFα, and mitigate oxidative stress by lowering peripheral superoxide anion levels [79,81].

Studies have shown that the gut microbiome of Multiple sclerosis (MS) patients differs from that of healthy individuals, with decreased abundance of certain beneficial bacteria, such as *Prevotella* and *Akkermansia*, and an increased abundance of pro-inflammatory bacteria, such as *Collinsella* and *Eggerthella*. Moreover, a high-fat diet, which is associated with dysbiosis (an imbalance of the gut microbiome), has been shown to exacerbate MS symptoms in animal models and humans [82].

Several studies have shown that the gut microbiome composition is altered also in Alzheimer’s disease (AD) patients, with increased levels of pro-inflammatory bacteria, such as *Escherichia coli*, and decreased levels of beneficial bacteria, such as *Bifidobacterium* and *Lactobacillus*. Additionally, high-fat diets have been shown to worsen cognitive decline and increase beta-amyloid accumulation in animal models of AD [83].

Research has revealed that the gut microbiome of individuals with Parkinson’s disease (PD) has an altered composition, characterized by reduced levels of beneficial bacteria like *Faecalibacterium* and *Prevotella*, and an increased presence of pro-inflammatory bacteria, such as Enterobacteriaceae. Furthermore, animal studies have demonstrated that consuming a high-fat diet can exacerbate motor symptoms and hasten disease advancement in PD [84].

A healthy diet, rich in fiber and beneficial nutrients, can promote a diverse and stable gut microbiome, which in turn, may reduce the risk of neurological diseases. Conversely, a high-fat diet, which can lead to dysbiosis and inflammation, may increase the risk of neurological diseases and worsen their outcomes. There is growing evidence that diet and the gut microbiome can impact the development and outcomes of depression, which is a common mental health disorder. Research has shown that the gut microbiome can influence the communication between the brain and the gut, known as the gut-brain axis, which plays a critical role in the regulation of mood, behavior, and cognitive function. Several studies have shown that the gut microbiome of individuals with depression differs from that of healthy individuals, with decreased levels of beneficial bacteria, such as *Bifidobacterium* and *Lactobacillus*, and increased levels of pro-inflammatory bacteria, such as *Escherichia coli* [14,35]. Moreover, dietary factors, such as a high-fat diet or a diet low in fiber, have been linked to dysbiosis and inflammation, which may exacerbate depressive symptoms [85]. Conversely, a healthy diet, rich in plant-based foods, whole grains, and beneficial nutrients, has been associated with a more diverse and stable gut microbiome, which in turn, may reduce the risk of depression and improve its outcomes [86]. Additionally, probiotics, which are live microorganisms that confer health benefits when consumed in adequate amounts, have been shown to improve depressive symptoms in some individuals [87].

Additionally, preclinical studies have shown that probiotics, which are live microorganisms that confer health benefits when consumed in adequate amounts, may improve cognitive function and reduce inflammation in animal models of schizophrenia [88]. Moreover, prebiotics, which are non-digestible food ingredients that promote the growth of beneficial bacteria in the gut, have been shown to improve cognitive function in individuals with schizophrenia [89].

It is worth mentioning here about, very low-calorie diets (VLCDs), very low-calorie ketogenic diets (VLCKDs), and very low carbohydrate diets (VLCarbDs) which are popular diets. These diets promote weight loss and improve metabolic health. However, recent studies have suggested that these diets may also have an impact on the composition of the gut microbiota. Studies have shown that VLCKDs may have a similar effect on gut microbiota composition and abundance as VLCDs, resulting in a decrease in *Firmicutes* and an increase in *Bacteroidetes.* Additionally, low-calorie diets have been associated with an increase in the abundance of *Akkermansia muciniphila*, a bacteria that is known to improve gut barrier function and reduce inflammation [90].

VLCKDs have been shown to reduce inflammation in the gut, which is often associated with several chronic diseases. A study by Paoli et al. [91] reported that VLCKDs reduced the expression of pro-inflammatory cytokines and increased anti-inflammatory cytokines in mice, indicating a decrease in inflammation in the gut. Similarly, a study by Lim et al. [92] found that a VLCKD reduced markers of inflammation in the gut of patients with obesity, which could potentially contribute to their overall health benefits. However, this beneficial change can be significantly enhanced if probiotics and prebiotics are supplemented during the diet.

## 3. The Significance of Adopting an Anti-Inflammatory Diet for the Prevention and Treatment of Neurodegenerative Disorders

### 3.1. Alzheimer’s Disease

Despite extensive efforts to prevent and treat Alzheimer’s disease (AD), it remains a global challenge, with the number of AD patients projected to reach 100 million worldwide by 2050 [91]. This multifactorial and heterogeneous neurodegenerative disease is characterized by a variety of irreversible behavioral changes [92]. Scientists have identified several risk factors for Alzheimer’s disease, and their association and overlap appear to increase the risk of developing the disease [92]. These risk factors include age, gender, and lifestyle factors such as cardiovascular health, alcohol consumption, social engagement, and sleep quality [93]. Research indicates that conditions such as depression and Down syndrome raise the likelihood of developing Alzheimer’s disease [94]. Individuals with Down syndrome exhibit Alzheimer’s disease symptoms 10–20 years earlier than the general population, with trisomy 21 being an undeniable risk factor for the disease [95,96]. Research has demonstrated that engaging in mentally and socially stimulating activities is linked with a decreased risk of developing Alzheimer’s disease [92]. Although the probability of developing Alzheimer’s disease increases with age, it is not an inevitable aspect of aging. Women account for nearly two-thirds of Alzheimer’s patients. The underlying mechanisms for these sex differences remain poorly understood. Some studies suggest that hormonal differences, particularly the effects of estrogen and testosterone, may play a role in the sex differences in neurological diseases [93,97,98,99] Estrogen has been shown to have neuroprotective effects, while testosterone may be neurotoxic [98,100,101]. However, the impact of these hormones on neurological diseases is complex and varies depending on the specific disease and stage of life. Other factors that may contribute to the sex differences in neurological diseases include differences in genetic susceptibility, immune function, lifestyle factors, and environmental exposure. Neurodegeneration in the form of extraneuronal neuritic plaques and neuronal death due to excessive production of amyloid B (AB) peptide are among the well-known hypotheses regarding the pathology of AD. Protein phosphatase 2A C (PP2Ac), as one of the tau protein phosphatases, is capable of dephosphorylating tau protein. However, the deactivation of PP2Ac hinders its ability to dephosphorylate tau protein, resulting in the eventual formation of neurofibrillary tangles [91]. Health problems such as lack of exercise, obesity, smoking (including passive smoking), high blood pressure, high cholesterol, and poorly controlled type 2 diabetes, which are risk factors for heart disease, can also increase the likelihood of developing Alzheimer’s disease [93,94]. Impaired brain energy metabolism is linked to progressive cognitive and functional decline in AD patients. Research suggests that AD patients exhibit lower levels of brain insulin signaling and fewer brain insulin receptors, which leads to brain insulin resistance [92]. What is more, the data obtained by positron emission tomography (PET) indicates a 20–25% deficiency in cerebral glucose metabolism. What is more, neurons indicate a diminished number of mitochondria. Worth mentioning is the fact that the mitochondria present in neurons show reduced citric acid cycle and respiratory chain activity, culminating in decreased energy production [93]. Several studies have examined the effect of diet on disease treatment, considering it a significant factor. Ketone bodies are a source of cellular energy, and a randomized crossover trial by Phillips and colleagues was conducted to assess the impact of the ketogenic diet on AD patients’ condition [93]. The study randomly assigned patients to a ketogenic diet or their usual diet supplemented with low-fat healthy-eating guidelines. During the dietary intervention, patients achieved a state of sustained physiological ketosis, with a mean beta-hydroxybutyrate level of 0.95+/−0.34 mmol/L over 12 weeks. Additionally, the patients demonstrated improvements in their within-individual scores on the ADCS-ADL (AD Cooperative Study–Activities of Daily Living) (mean increase of +3.13+/−5.01), QOL-AD (Quality of Life in AD) (mean increase of +3.37+/−6.86), and ACE-III (Addenbrookes Cognitive Examination–III). However, the increase in ACE-III values was not significant (+2.12+/−8.70). What is more, the cardiovascular risk factors were low [93].

It was not the first evaluation of the influence of fats on patients with AD. In 2018, Orti with scientists from Spain evaluated the influence of the Mediterranean diet enriched with coconut oil on AD patients [102]. In the study conducted by Ota and colleagues [103], AD-diagnosed patients were administered a medium-chain-triglyceride (MCD)-based ketogenic diet consisting of 50 g of ketogenic formula (Ketonformula) containing 20 g of MCTs. Meanwhile, patients in the experimental group were given a Mediterranean diet enriched with coconut oil for a duration of 21 days. The administration of the Mediterranean diet with coconut oil was reported to improve episodic and temporal orientation, as well as semantic memory, with a more significant effect observed in women with mild to moderate states [102]. According to the findings, patients’ performance on the digit-symbol coding test and immediate logical memory test showed a significant improvement compared to their baseline scores. Additionally, both their immediate and delayed logical memory tests also demonstrated a notable increase [104].

Fortier et al. also found comparable outcomes, stating that mild cognitive impairment patients exhibited enhanced cognitive abilities after 6 months of consuming a ketogenic beverage [105]. In another study, Brandt et al. [102] demonstrated the advantageous effects of the Modified Atkins Diet (MAD) for patients in the early phases of AD who experienced mild cognitive impairment and memory loss. There was observed a significant improvement in psychomotor activity and memory in early AD patients [102]. Akbari and colleagues conducted a randomized controlled trial in 2019 to evaluate the effects of a ketogenic diet on cognitive function and inflammatory markers in patients with Alzheimer’s disease (AD) [41]. The study included 80 participants with AD who were randomly assigned to either a ketogenic diet or a usual diet. After 12 weeks, the researchers found that the ketogenic diet group had significant improvements in cognitive function compared to the usual diet group, as measured by the Mini-Mental State Examination (MMSE). Additionally, the ketogenic diet group had significantly lower serum levels of IL-6 and TNF-alpha, indicating a reduction in inflammation.

The Mediterranean diet is recognized for its health benefits, featuring high consumption of fruits, vegetables, whole grains, legumes, and nuts, moderate intake of fish, poultry, and alcohol (especially red wine with meals), and low intake of red and processed meats. Its use enhanced the cognitive function like as episodic memory which was established in a number of research conducted among others by Loughrey et al., 2017 [106]; Katsiardanis et al., 2013 [107]; Kesse-Guyot et al., 2013 [108]; Zbeida et al., 2014 [109]; Anastasiou et al., 2018 [110]; Galbete et al., 2019 [109]; Roberts et al., 2010 [111]; McGrattan et al., 2019 [36]. According to research findings, adherence to the Mediterranean diet has been linked to improved cognitive performance, reduced risk of cognitive decline and impairment, and a lower incidence of Alzheimer’s disease [110]. In a study conducted by Hoscheidt et al., the impact of both Mediterranean and Western diets on Alzheimer’s disease biomarkers was assessed [112]. In a randomized trial, patients with mild cognitive impairment were randomly assigned to receive either a Mediterranean diet or a Western diet. The Western diet was high in saturated fat, glycemic index, and sodium, while the Mediterranean diet was isocaloric and characterized by a high intake of fruits and vegetables, whole grains, legumes, and nuts, a moderate intake of fish, poultry, and alcohol, especially red wine with meals, and a low intake of red and processed meats. Surprisingly, the researchers found that in healthy individuals, the Mediterranean diet increased CSF amyloid Beta (AB) levels, while the Western diet decreased them. Additionally, in the group of patients with normal cognitive function, the Western diet decreased cerebral perfusion, while the Mediterranean diet increased it. However, in the group of patients with impaired cognitive functions, the opposite reaction to that described in healthy subjects was observed (amyloid Beta (AB) level in CSF increased after a Western diet and decreased after the Mediterranean diet) [113].

Shinto et al. [114] evaluated the effects of ω-3 supplementation alone and in combination with lipoic acid (LA) on oxidative stress and cognitive function in AD patients. The study primarily measured the peripheral F2-isoprostane level, which reflects lipid oxidation, and assessed cognitive function using AD assessment scale-cognitive subscale (ADAS-cog), mini-mental state examination (MMSE), and functional ability (activities of daily living instrumental activities of daily living (ADL/ADL). After 6 and 12 months of supplementation, the percentage of DHA and EPA in the red blood cell membrane increased in patients who received fish oil. The group of patients who received ω-3 + LA had higher serum LA levels compared to other groups. However, there was no significant difference in F2-isoprostane levels at 6 and 12 months. Both time points were similar. Scientists report that it could be caused by the difficulties in interpretation of the treatment effects because of the high level of significantly higher baseline F2-isoprostane levels in the ω-3 group. The ADAS-cog evaluation showed no significant difference between the ω-3 + LA, ω-3 groups and the placebo group. However, the group receiving ω-3 + LA showed less decline in MMSE and IADL, while the ω-3 group exhibited less decline in IADL. In conclusion, Shinto et al. reported that the combination of ω-3 + LA was able to slow down cognitive and functional decline in AD over 12 months, but further evaluation is required [114].

Sun and co-workers conducted studies with the aim of analyzing the relationship between malnutrition and hyperhomocysteinemia in AD patients and the effect of diet intervention with betaine [94,115]. A total of 97 patients diagnosed with AD participated in the trial, and their nutritional status was assessed using the short-form mini-nutritional assessment (MNA-SF). In addition, biochemical parameters including plasma homocysteine (HCY) levels, hyperphosphorylation, synaptic proteins, and blood inflammatory factors were measured through enzymatic cyclic assay, western blot, and ELISA. As a result, malnutrition was reported in a larger population of AD patients which was related to high levels of HCY [94]. Betaine administration led to a decrease in the phosphorylated Tau protein level and an increase in PP2Ac activity. Additionally, betaine supplementation was found to inhibit the accumulation of A-Beta. The concentration of proinflammatory cytokines IL-1β and TNF-α was significantly lower compared to the non-treated group [94].

The ADAS-cog test also confirmed that the use of betaine improves cognitive function in AD patients [112]. This study indicates how significant an impact diet has on the treatment of AD. It is clearly exposed that malnutrition worsens AD patient state. The health-promoting properties of walnuts, including their antioxidant and anti-inflammatory effects, have been widely recognized [116]. To investigate the potential cognitive benefits of walnut consumption, Sala-Vila et al. conducted a randomized controlled trial in cognitively healthy elderly individuals. Surprisingly, the 2-year walnut supplementation did not have a significant effect on cognition. However, fMRI analysis suggested that a nut-rich diet may delay cognitive decline in high-risk subgroups [117]. Furthermore, certain dietary supplements can also interact with drugs used in the treatment of neurodegenerative diseases. For example, St. John’s wort, a popular herbal supplement used for the treatment of mild-to-moderate depression, can induce the liver enzyme CYP3A4, which metabolizes many drugs, including some used to treat Alzheimer’s disease [118]. As a result, taking St. John’s wort with Alzheimer’s medications, such as donepezil, galantamine, or rivastigmine, can decrease their efficacy and lead to treatment failure [117]. Therefore, patients taking medications for Alzheimer’s disease should be advised to consult their healthcare provider before taking any dietary supplements or herbal remedies.

### 3.2. Parkinson’s Disease

Parkinson’s disease, a neurodegenerative disorder, affects a majority of individuals over 50 years of age and is projected to affect 8.7–9.3 million people over 50 by 2030. The disease is characterized by bradykinesia, tremor, and plastic rigidity, which arise from the loss of dopaminergic neurons in the substantia nigra. Additionally, the disease is associated with non-motor symptoms that contribute to overall disability. Parkinson’s disease is distinguished by neuronal loss in the substantia nigra, striatal dopamine deficiency, and intracellular inclusions containing alpha-synuclein aggregates [119,120,121,122]. Recent studies suggest that the development of PD involves multiple factors, including impaired mitochondrial function, oxidative and nitrative stress, the buildup of misfolded proteins, and dysfunction of the ubiquitin-proteasome system. Furthermore, genetic factors also play a significant role in the pathogenesis of this disease [120]. Some studies have suggested that certain dietary factors may influence the risk of developing PD. For instance, a diet rich in antioxidants, such as vitamin C, vitamin E, and beta-carotene, has been associated with a lower risk of PD [123]. Mediterranean diet, which is high in plant-based foods, fish, and olive oil, has been linked to a reduced risk of Parkinson’s disease [124]. Conversely, a diet high in saturated fat and cholesterol has been linked to an increased risk of PD [122]. Therefore, patients with PD may benefit from dietary counseling to optimize their nutrition and reduce their risk of disease progression.

As mentioned in previous parts of this manuscript, the relationship between the brain and the gut microbiome in the context of health, and chronic central nervous system diseases has been increasingly appreciated in recent years.

Tamtaji and co-workers (2018) have shown that 12 weeks of use of probiotics (containing strains of Lactobacillus acidophilus, Bifidobacterium bifidum, Lactobacillus reuteri, and Lactobacillus ursi) by Parkinson’s disease patients enhanced their metabolic state [125]. It noted a decrease in insulin resistance and CRP protein as well as malondialdehyde levels and as well as increased glutathione levels [125]. Worth mentioning, food sources rich in probiotics are e.g., yogurt [126], kefir [127], kimchi (which contains *Lactobacillus kimchi spp*.) [128], sauerkraut as well as pickles [128]. Regarding the characteristics of the Mediterranean diet, which includes a huge number of vegetables and fruits, and, wine, fruits it is acknowledged that it possesses antioxidant activity and can be used to enhance the treatment of or/and prevent central nervous system diseases. There is evidence promoting its use in the prevention and treatment of PD [123]. In 2020, Paknahad et al. evaluated how the Mediterranean diet (MeD) affected the anti-oxidative status and clinical condition of individuals with PD [129]. Results confirmed the previous evaluation, indicating a meaningful decrease from baseline in all UPDRS composites. Surprisingly, except for motor examination (change: 0.3 for MeD and −0.6 for the control group). Scores of the mental, behavioral, and mood UPDRS scale decreased (change −1.02) in a group of MeD-treated patients and increased (change 0.03) in a control group. The increase in daily living activity levels was also noted. It is worth indicating, in fact, that activity of daily living significantly increased (change in UPDRS scale for MeD: −3.4). It was also acknowledged that serum total antioxidant capacity (TAC) also increased after the Mediterranean diet intervention which also confirmed its health-promoting activity [130]. Several studies have investigated the effects of the ketogenic diet on PD patients, similar to those exploring its impact on AD patients. Vanitallie et al., (2005) conducted research on PD subjects at the Beth Israel Medical Center’s Movement Disorders Clinic, using a hyper ketogenic diet (HKD) for 28 days. Their findings indicated that the HKD intervention improved resting tremors, freezing, gait, mood, and energy levels [131].

In 2020, Paknahad et al. conducted a randomized controlled trial to investigate the effects of a Mediterranean diet on inflammatory markers and motor function in patients with Parkinson’s disease (PD) [44]. The study included 80 participants with PD who were randomly assigned to either a Mediterranean diet or a control diet group. After 3 months, the researchers observed significant reductions in inflammatory markers and greater improvements in motor function in the Mediterranean diet group compared to the control diet group. The motor function changes were measured using the Unified Parkinson’s Disease Rating Scale (UPDRS). These findings suggest that a Mediterranean diet may serve as an effective intervention for reducing inflammation and improving motor function in individuals with Parkinson’s disease.

A study from 2018 compared the effects of a low-fat versus ketogenic diet in PD patients. Patients had an 8-week diet intervention [129]. Changes in the MDS UPDRS scale were examined at the beginning of the trial and after 8 weeks. As a result, nonmotor daily living experiences in UPDRS scores decreased in both groups, although the change was more significant in the ketogenic group (change 4.09 in the ketogenic group vs. 0.99 the in low-fat group). Similar results were obtained when motor daily living experiences were measured. Change in the ketogenic group equals 3.13 and the change in a low-fat group is 1.33. The low-fat diet group showed better results in the motor examination (with a change of −8.58) compared to the ketogenic diet group (with a change of −6.27) in part 3 of the study. However, patients who received a ketogenic diet had greater improvement in motor complications (with a change of 1.56) compared to the low-fat diet group (with a change of 0.79). Both the ketogenic diet group and the low-fat diet group showed significant improvements in motor and nonmotor symptoms. However, the ketogenic diet group showed greater improvements in nonmotor symptoms [130]. Philips and colleagues conducted a randomized controlled trial in 2018 to investigate the effects of a ketogenic diet on inflammatory markers in patients with Parkinson’s disease (PD) [42]. The study involved 44 participants with PD who were randomly assigned to either a ketogenic diet or a usual diet. The researchers found that the ketogenic diet group had significant reductions in all inflammatory markers compared to the usual diet group after 8 weeks. This suggests that the ketogenic diet may have the potential as an intervention for reducing inflammation in individuals with Parkinson’s disease. However, it is important to note that this study had a small sample size and a short duration, which limits the generalizability of the results. Further research is needed to confirm these findings and explore the potential benefits and risks of a ketogenic diet for individuals with Parkinson’s disease.

Zhang and colleagues conducted a prospective cohort study in 2017 to investigate the association between dietary patterns and the risk of Parkinson’s disease (PD) [45]. The study included 198,584 participants who were followed for 10 years to monitor incident cases of PD. The researchers found that higher adherence to a prudent dietary pattern was associated with a lower risk of PD.

In a separate study by Gao and colleagues in 2007, a cross-sectional design was used to evaluate the association between Dietary patterns, inflammation, and risk of PD [46]. The researchers found that the Mediterranean diet score was associated with lower levels of inflammatory markers, including tumor necrosis factor-alpha (TNF-alpha) and interleukin-6 (IL-6), and better motor function in patients with PD. Diet plays also an important role in the pharmacological treatment of Parkinson’s disease (PD). For example, levodopa, the most effective medication for managing the motor symptoms of PD, is absorbed better on an empty stomach [132]. However, some evidence suggests that the drug may be more effective when taken with a low-protein diet [133]. This is because high-protein meals can compete with levodopa for absorption in the gut and reduce its effectiveness. Therefore, patients taking levodopa should be advised to follow a low-protein diet or to separate the timing of their protein intake from their medication schedule to ensure optimal efficacy.

### 3.3. Huntington’s Disease

Huntington’s disease (HD) is a neurodegenerative disease with a genetic background for which there is no effective treatment.

The main symptoms are an uncontrolled movement of the head, face, arms, and legs. This disease also causes a decline in concentration, memory, and ability to plan [133,134]. The onset of Huntington’s disease symptoms can occur at any age, although it is more common in individuals between the ages of 30 and 40, and when it occurs at a younger age, the disease may progress more rapidly. Studies have revealed that oxidative stress, which leads to a deficiency in antioxidant systems and inflammation, plays a critical role in the initiation and advancement of HD. Moreover, there is strong evidence indicating a connection between immune activation and brain damage induced by proapoptotic agents [133]. In addition, imaging studies utilizing positron emission tomography have demonstrated that inflammation precedes the onset of symptoms in genetically diagnosed HD patients [134]. Animal models of Huntington’s disease have also shown that treatments targeting tissues and organs outside the CNS can modulate synapse loss and behavioral changes [135]. Furthermore, links between peripheral biology and neurodegeneration have been discovered in other chronic neurodegenerative diseases, suggesting that targeting these peripheral mechanisms may provide new therapeutic avenues [40,136].

Apart from behavioral and psychiatric disorders, patients struggle with eating and swallowing (dysphagia) with the disease progression. Cognitive disorders may prevent the person from choosing appropriate foods with lots of calories and easy to eat. People with HD frequently have lower-than-average body weight which on the one hand may be related to diet and to the other biochemical changes caused by Huntington’s disease [137]. As there is no efficient therapy for HD scientists are forced to seek daily solutions which can slow up HD development. It seems that a properly selected diet can improve the physical performance and mental health of HD patients [137,138].

Moghaddam and colleagues found that a diet enriched with elderberry improved motor function and limited oxidative stress and inflammation in a rat model of Huntington’s disease. It was noted that the elderberry diet significantly decreased the level of caspase-3 and TNF-alpha and also improved striatal antioxidative capacity through an increase in GSH and the reduction of ROS [135].

Rivadeneyra et al. found that HD patients who followed a moderate Mediterranean diet (MeDi) had improved mobility and quality of life, as well as less psychiatric impairment [139].

Marder et al. showed that higher dairy consumption and higher caloric intake (associated with low adherence to MeDi) are associated with a higher risk of phenoconversion in HD patients, which is unfavorable [137,140]. Patients with severe HD were reported to have a higher caloric intake and BMI than those with mild-moderate HD, as described by Cubo et al. [141]. However, patients with medium-high adherence to the Mediterranean diet had a similar caloric intake, BMI, TFC, PBA, and motor and cognitive UHDRS scores compared to those with low adherence [138]. Patients with high adherence to MeDi had a higher quality of life. What is interesting is that patients with advanced HD had a higher intake of vitamins soluble in water and minerals [138].

Based on available knowledge it can be concluded that a ketogenic diet has also a favorable influence on the central nervous system. Scientists from the United States [142] during the use of a ketogenic diet in transgenic HD mice (R6/2 1J) did not observe negative effects on any behavioral parameters (such a locomotor activity, coordinator, or working memory). What’s more, it was noted that body weight reduction in transgenic mice can be crucial, regarding the fact that weight loss is a hallmark feature of HD [140].

Some studies have suggested that a high-fat diet can exacerbate HD symptoms and accelerate disease progression in animal models, possibly due to increased oxidative stress and inflammation [143]. Therefore, reducing dietary fat intake and increasing the consumption of antioxidants may have a protective effect against HD. In addition, some evidence suggests that a ketogenic diet, which is high in fat and low in carbohydrates, may improve motor function and cognitive performance in patients with HD, possibly by enhancing mitochondrial function and reducing oxidative stress [143].

### 3.4. Multiple Sclerosis

Multiple sclerosis (MS) is one of the most common demyelinating illnesses characterized by recurring neurological episodes caused by widespread demyelinating lesions in the central nervous system. These lesions cause serious neurological impairments over time [144].

The most prevalent form of this disease is recurrent relapses (RR MS), in which each occurrence of new pathological abnormalities discovered via MRI corresponds to a new clinical episode. Around 80% of cases of this disease start by this form. A chronic progressive variant (PP MS) is also distinguished, in which symptoms progress and worsen with time. MS becomes secondary chronic after several years of RR MS progression [145]. There is also a moderate form of MS that affects about 15% of all patients but does not produce major neurological damage within 15 years of onset [144].

The malignant type, known as Schilder’s illness, is distinguished by rapid progression and the emergence of severe neurological symptoms, even within weeks of diagnosis. The schilder disease causes breathing and consciousness problems, and it can even result in death [144]. The etiology of the disease remains incompletely understood, but it is believed to be a complex disorder involving genetic susceptibility, immune system elements, and environmental factors like obesity, smoking, as well as bacterial and viral agents (although no specific pathogens have been identified so far) [142]. Multiple sclerosis is more commonly observed among young adults, particularly women, typically between the ages of 20 and 40, while early onset accounts for 3–5% of all cases before the age of 16 and late onset accounts for 3.4–12.7% of cases beyond the age of 50 [145]. Pharmacotherapy in MS is reduced to the symptomatic treatment of attacks and slows down the further development of the disease. Interferon beta can be administered intramuscularly or subcutaneously, or glatiramer acetate can be administered. Glucocorticosteroids are largely used to treat MS relapses because they limit the inflammatory process [144]. Epidemiological studies have found a relationship between food and the occurrence of MS. Nutrition has been considered one of the environmental elements implicated in MS etiology [146]. As it was comprehensively described in the previous part of the manuscript, an anti-inflammatory diet consisting of a large number of fruits, vegetables, low-fat products, and a large number of dairy products with probiotics may have a significant impact on the progression of changes in the central nervous system. In the evaluation conducted by Mousavi-Shirazi-Fard et al. [146] patients with MS received an anti-inflammatory diet based on a large number of vegetables and fruits [40].

Patients were advised to make dietary substitutions, such as switching from white rice and bread to brown varieties and replacing high-fat dairy products with low-fat probiotic alternatives. Legumes, healthy fats like extra-virgin olive oil, canola oil, and nuts, as well as spices like ginger, cinnamon, and turmeric, were recommended. Moderate amounts of white or green tea and dark chocolate were also encouraged, while red meat and eggs were limited to 1–2 times per week. On the other hand, refined carbohydrates, sucrose-containing products, processed and fried foods, and animal fats were discouraged. Results showed that adhering to this anti-inflammatory diet significantly improved the Modified Fatigue Impact Scale (MFIS) and physical and mental components of the Multiple Sclerosis Quality of Life (MSQoL-54) in MS patients, along with increased levels of the anti-inflammatory cytokine interleukin 4 (IL-4). The applied nutritional scheme will not significantly affect the level of IL-17 as well as the high sensitivity C-Reactive Protein level [146,147].

Bock et al., (2019), the impact of a ketogenic diet (KD) and caloric restriction (CR) on serum neurofilament light chain level (sNfL) in MS patients was investigated. sNfL is a promising biomarker of neuroaxonal damage [148]. The study revealed that the KD group had significantly lower sNfL levels at 6 months compared to the control group (Common diet CD), indicating the potential neuroprotective effect of KD in MS [148]. Katz Sand and colleagues during the pilot study established that the use of the Mediterranean diet reduced fatigue measured using the Neurological Fatigue Index MS (NFI-MS) in females with diagnosed MS [40]. During the 6 months period, the non-intervention group increased by about 2.2 points on average whereas the intervention group decreased by −4.6 points of NFI-MS. After the period of 6 months, the non-intervention group increased by about 4.8 and the intervention group decreased by about 7.4 points. The diet intervention also reduced disability measured by the Expanded Disability Status Scale (EDSS). Over the 6 months, the dietary intervention group revealed a statistically significant decrease in the trajectory of EDSS scores. However, scientists implied that further work on the influence of MeDi on MS is needed [149]. In conclusion, MeDi gives hope for its use as a supportive treatment of MS, improving the quality of life. Platero and co-workers noted that a diet rich in coconut Oil and Epigallocatechin Gallate (EGCG) decreased anxiety and improved state functional capacity in MS patients which was correlated with a decrease in the level of IL-6 [150]. In a randomized controlled trial, Mousavi-Shirazi-Fard and colleagues investigated the effects of an anti-inflammatory diet on inflammatory markers in patients with multiple sclerosis (MS) [40]. The study included 100 participants with MS who were randomly assigned to either an anti-inflammatory diet or a usual diet group. The researchers measured serum levels of inflammatory markers, such as CRP, IL-4, and IL-17, at the beginning and end of the study period. The study found that the anti-inflammatory diet group had significant reductions in some inflammatory markers compared to the usual diet group. These findings suggest that an anti-inflammatory diet could potentially reduce inflammation in individuals with MS.

As the use of antioxidants is acknowledged to be helpful in the treatment of MS, scientists from Spain evaluated how a low-fat diet in the combination with antioxidant supplementation (e.g PUFA, Vitamins, Quercetin, Coenzyme Q, Lipoic/linoleic acid) prepossess on biochemical markers of inflammation. A low-fat diet combined with antioxidant supplementation was found to decrease C-reactive protein levels, as well as other inflammatory markers such as isoprostane 8-iso-PGF2alpha and interleukin IL-6. In addition, there was a significant increase in catalase activity in the blood of MS patients, according to the study’s findings [148]. As we know, catalase is a powerful antioxidant enzyme responsible for removing hydrogen peroxide produced in pathological processes such as inflammation [149]. In addition, some studies have suggested that vitamin D, which can be obtained through diet and sun exposure, may enhance the efficacy of pharmacological treatments for multiple sclerosis (MS) [150]. A study by Ascherio and Munger (2017) found that higher levels of vitamin D were associated with a lower risk of MS and a slower progression of the disease [151]. Moreover, vitamin D supplementation has been shown to enhance the effectiveness of immunomodulatory drugs used to treat MS, such as interferon-beta [152]. These findings suggest that vitamin D may be a promising adjunct therapy to enhance the pharmacological treatment of MS [15].

## 4. Anti-Inflammatory Diet and Its Role in Mental Health

### 4.1. Depression

Depression is a common mental disorder that is marked by persistent sadness, a depressed mood, a decrease in physical activity, and a loss of interest in anything [153]. In fact, depression is one of the most frequently diagnosed central nervous system disorders. In 2017, depressive disorders ranked as the second highest-diagnosed mental disorder, following anxiety disorders, according to estimations by the Institute for Health Metrics and Evaluation (IHME). Depression affected 20.7 million individuals, or 4.2% of the population, in the European Union, and 4.5 million people, or 1% of the population, in the same year [153].

The outbreak of the COVID-19 pandemic in 2019 has created a good environment to increase mental health problems in the general population. According to reports from international institutions, the number of cases of depression will rise in the future [154]. At present, it is thought that a combination of factors, including genetic and personality predisposition (such as low self-esteem or high stress), as well as environmental factors like repeated violence, neglect, abuse, and poverty, contribute to the development of this condition. The role of diet in the development of an inflammatory state in depression has recently been identified as a key contributor to its prevalence [155]. Dietary habits that are abundant in pro-inflammatory elements are linked with elevated levels of inflammatory markers, as per research findings [156,157].

In their study, Wang et al. found that a pro-inflammatory diet, characterized by a higher DII score, was linked to a greater risk of depression, especially in women [157]. The study revealed that those with the highest DII score (suggesting a greater pro-inflammatory dietary potential) had a 23% higher likelihood of experiencing depression. A similar outcome was observed in the longitudinal cohort study conducted by Shivappa and colleagues, who investigated the association between DII and the emergence of depressive symptoms [158]. Over the course of eight years, 837 people (310 men and 527 women) experienced incidental depression symptoms. Patients with a pro-inflammatory diet had a 24% higher risk of developing depressive symptoms than patients with an anti-inflammatory diet [159]. In another study, Shivappa et al. conducted a study focusing on the potential link between the inflammatory impact of diet and depression risk among adolescent females in Iran [158]. Females who ate the pro-inflammatory diet had higher depression scores and were 3.96 times more likely to have moderate depression than females who followed an anti-inflammatory diet. This finding suggests that modifying the inflammatory potential of one’s diet could be a viable technique for preventing depression [156,157]. Numerous studies present molecular mechanisms underlying the beneficial effects on the mental health of specific nutritional components [153]. It was found that diet may have an impact on the modulation of inflammation, oxidative stress, epigenetics, mitochondrial dysfunction, the gut microbiome, tryptophan-kynurenine metabolism, the HPA axis, and neurogenesis [157,158,159,160,161]. The study, conducted by Lai et al., (2014), was a controlled trial that aimed to investigate the impact of dietary patterns on depression, anxiety, and inflammatory markers [47]. The study included adults with moderate to severe depression who were randomly assigned to either the DASH diet or the control diet. The results showed that the DASH diet group had significant reductions in depression severity and anxiety scores compared to the control group. Additionally, the DASH diet group had lower levels of inflammatory markers such as CRP, IL-6, and TNF-alpha.

In turn, El-Mallakh et al., (2020) investigated the effects of a ketogenic diet on treatment-resistant depression, anxiety, and inflammatory markers [48]. The study included patients with treatment-resistant depression who were randomly assigned to either the ketogenic diet or the control diet. The results of the study demonstrated that the ketogenic diet group had a significant reduction in depression severity and anxiety scores compared to the control group. Furthermore, the participants on the ketogenic diet had lower levels of inflammatory markers, including CRP and IL-6, compared to the control group.

Both studies suggest that dietary interventions may have a positive impact on depression, anxiety, and inflammatory markers. The DASH diet and ketogenic diet were both effective in reducing depression and anxiety symptoms, as well as lowering inflammatory markers. However, it is important to note that these studies were conducted in relatively small sample sizes and over a relatively short period. Further research is needed to confirm these findings and to investigate the long-term effects of dietary interventions on mental health and inflammation.

According to Godos et al., (2020), consuming plant-based foods, such as fruits, vegetables, legumes, and whole grains, which contain anti-inflammatory and antioxidant components, has been shown to possess neuroprotective properties [161]. Similarly, certain polyunsaturated fatty acids (PUFA) found in fish, such as omega-3, and monounsaturated fatty acids (MUFA) from extra virgin olive oil are also known for their anti-inflammatory benefits, which can enhance cognitive function [158]. In a review study conducted by Ljungberg, strict adherence to dietary guidelines that involve avoiding processed foods and consuming an anti-inflammatory diet, rich in magnesium and folic acid, as well as various fatty acids, was linked to a significant reduction in the severity of depression symptoms [162]. Emerging research suggests that altering the composition of the gut microbiota through probiotic supplements could be a potential adjuvant treatment option for people suffering from serious depression (MDD) [162]. The existing evidence suggests that probiotics may lessen depression symptoms by modulating inflammation. Considering these reports, it seems that probiotics may be very promising as a primary, supplementary, or preventative treatment for depression [161,162,163].

Diet and its ingredients may play a role in the effectiveness of pharmacotherapy for depression. The omega-3 fatty acids, which are found in fish, nuts, and seeds, have been shown to have antidepressant effects and enhance the efficacy of antidepressant medications, possibly by modulating neurotransmitter systems and reducing inflammation [164]. In addition, some evidence suggests that a Mediterranean-style diet, which is high in fruits, vegetables, whole grains, legumes, nuts, and olive oil, may be associated with a lower risk of depression and improve response to antidepressant treatment, possibly due to its anti-inflammatory and antioxidant properties [163,164,165,166,167]. On the other hand, a diet high in processed or refined foods, such as sugar and saturated fat, may have a detrimental effect on mood and increase the risk of depression, possibly by promoting inflammation and oxidative stress [164]. Therefore, dietary interventions may have a complementary role in the treatment of depression, and patients may benefit from nutritional counseling and supplementation.

### 4.2. Schizophrenia

Schizophrenia (SZ) is a chronic disease that is classified as a psychotic disorder- a condition characterized by a pathologically altered, inadequate perception, experience, and evaluation of reality. The symptoms of this disorder comprise enduring hallucinations, delusions, disorganized speech, disorganized or catatonic behavior, and negative symptoms such as emotional flatness, apathy, and reduced speech. Although schizophrenia is not as common as other mental illnesses (it affects about 1% of the population), it can nevertheless cause disability and a serious financial and social burden on patients’ families [168]. Individuals with psychosis experience a significant decline in their capacity to objectively and reasonably assess themselves, their surroundings, and their interactions with others. There are various types of schizophrenia. Paranoid, hebephrenic, catatonic, simple, residual, and undifferentiated schizophrenia are distinguished based on the prevailing symptoms [169]. Schizophrenia has a multifactorial cause and a complex presentation. Advances in neuroscience have identified key circuits in the development of positive, negative, and cognitive symptoms, particularly those involving frontal, temporal, and mesostriatal brain regions [168,170]. The pathophysiology of schizophrenia remains partially elusive, and antipsychotics presently in use have notable shortcomings [168]. Recent evidence indicates that inflammation may play a critical role in the pathophysiology of schizophrenia [169]. The anti-inflammatory effects of individual foods and dietary patterns are well established. Hence, certain foods and dietary regimes can either reduce or exacerbate inflammation. Observational studies have demonstrated that consuming a diet rich in fruits, vegetables, legumes, and olive oil, like the Mediterranean diet, can potentially safeguard against mental health conditions. Conversely, diets high in saturated fats and refined carbohydrates, such as the Western diet, have been associated with an elevated likelihood of mental disorders. The development of schizophrenia has been associated with heightened levels of pro-inflammatory cytokines and microglia activation [170,171,172,173]. After considering the expenses incurred in treating schizophrenia, as well as the unhealthy eating habits of individuals with this condition that can result in obesity and metabolic syndrome, researchers have concluded that including anti-inflammatory nutrients or foods as part of a well-balanced diet can be advantageous in the long term to reduce the need for medication and its adverse effects [174].

Jahrami et al. confirmed in their studies a positive correlation between the dietary inflammatory index (DII^®^) and schizophrenia [175]. Accumulating evidence points to the important role of signaling between the central nervous system (CNS) and the gut nervous system (“microbiome-gut-brain (MGB) axis”) in schizophrenia [176]. It turns out that dietary inulin could be a unique technique for the treatment of schizophrenia by regulating gut microbiota. This fructan is a non-digestible carbohydrate that has been part of our everyday diet for generations. Inulin is found naturally in several vegetables and fruits, primarily in the tubers and roots of lily and *Asteraceae* plants. Onion, garlic, Jerusalem artichoke, dahlia, asparagus, artichoke, dandelion, and burdock are all good sources of inulin. Inulin administration by gavage improved aberrant behaviors (locomotor hypoactivity, anxiety disorders, and depressive behaviors, as well as impaired learning and spatial recognition memory), and effectively reduced neuroinflammation and neuronal damage in mice. Furthermore, inulin increases gut integrity and permeability, which is favorable [177]. Inulin enhanced the number of strains of *Lactobacillus* and *Bifidobacterium*, which were negatively correlated with the level of 5-hydroxytryptamine and inflammatory cytokines and positively correlated with brain-derived neurotrophic factor (BDNF) [177]. Various studies have investigated the impact of supplementing with omega-3 polyunsaturated fatty acids, vitamin D, B vitamins (including B6, folic acid, and B12), vitamin E, and carotenoids on the symptoms associated with distinct stages of schizophrenia [178,179,180]. Current evidence suggests that supplementing with omega-3 PUFAs, vitamin D, and vitamin B may be beneficial in treating schizophrenia, although further research is necessary. Additionally, vitamin E supplementation has the potential to enhance the efficacy of other nutrients, like vitamin C and omega-3 PUFAs, in reducing schizophrenia symptoms [178].

Due to the inadequate effectiveness of existing antipsychotic drugs, especially with respect to negative symptoms, cognitive impairments, and day-to-day functioning, in addition to their considerable extrapyramidal and metabolic adverse effects and high expense, alternative treatment strategies are undoubtedly necessary [181,182,183]. However, due to the heterogeneity of the underlying pathophysiology in the condition, the likelihood of a ‘one size fits all’ nutritional intervention for the treatment of schizophrenia is slim. A systematic review found that supplementing with certain nutrients, such as omega-3 fatty acids, B vitamins, and antioxidants may improve cognitive and clinical outcomes in patients with schizophrenia, possibly by reducing oxidative stress and inflammation [179]. In addition, some evidence suggests that a Mediterranean-style diet, which is high in fruits, vegetables, whole grains, legumes, nuts, and olive oil, may be associated with a lower risk of schizophrenia and improved cognitive function in patients with schizophrenia, possibly due to its anti-inflammatory and antioxidant properties [180]. On the other hand, a diet high in sugar and saturated fat may harm cognition and increase the risk of schizophrenia, possibly by promoting inflammation and oxidative stress [181]. Therefore, a personalized medicine strategy, incorporating nutritional therapy as a significant component, may prove to be a more advantageous approach for managing individuals with schizophrenia [181,182,183,184,185].

### 4.3. Bipolar Disorder

Bipolar disorder (BD), which is also referred to as manic-depressive illness, psychosis, or cyclophrenia, is distinguished by periods of depression, mania/hypomania, or a blend of the two. The second most common cause of psychological impairment is a bipolar illness. It typically begins at a young age (before the age of 35), which, when combined with a high recurrence of symptoms, contributes to serious, negative consequences in all aspects of the patient’s life (social, family, professional functioning, economic condition) [186]. Bipolar disorder is considered a set of affective illnesses–mood disorders defined by recurring depressive, manic, hypomanic, or mixed episodes of varying severity, course, and response to therapy [187]. Bipolar disease is one of the biggest causes of disability in young people, causing cognitive and functional impairment as well as increased mortality, including suicide. It affects more than 1% of the global population, regardless of nationality, ethnicity, or socioeconomic level. In clinical practice, an accurate diagnosis of bipolar disorder is said to be challenging because the outset is typically a depressive episode that looks identical to unipolar depression [188]. Studies have revealed that BD is linked to a high prevalence of inflammatory medical comorbidities, including autoimmune illnesses, chronic infections, cardiovascular disease, and metabolic disorders. Chronic low-grade inflammation has been associated with BD, with pro-inflammatory cytokine levels rising during disease exacerbations. A connection between BD and immunological dysfunction has been established, and key processes in the pathomechanism of BD include cytokine-induced monoamine changes, increased oxidative stress, pathological microglial over-activation, HPA axis over-activation, modifications to the microbiome-gut-brain axis, and sleep-related immunological changes [189]. These offer novel treatment options for BD.

Furthermore, anti-inflammatory drugs have been demonstrated to be effective in BD patients. Rosenblat et al. investigated the impact of anti-inflammatory drugs in bipolar depression. The quantitative review includes eight randomized controlled trials with the following agents: omega-3 fatty acids, nonsteroidal anti-inflammatory medications, *n*-acetylcysteine, and pioglitazone. The pooled effect size of all adjunctive medicines on depression was moderate, indicating an overall effect of these drugs on BD patients [189,190].

The microbiome is a fast-evolving scientific frontier with implications for mental disorders. The gut microbiota interacts bidirectionally with the central nervous system via the gut-brain axis and substances that may alter the host’s metabolism, such as short-chain fatty acids like butyrate. Understanding the participation of the gut microbiota in bipolar disorder (BD) may lead to the identification of new illness markers and treatment methods. Low diversity and dysbiosis in terms of *Faecalibacterium* and *Bacteroides* abundance may characterize BD in both a trait-like and a state-dependent manner. The reduced richness and butyrate synthesis also promote inflammation, which may be an unnoticed component of the pathophysiology of BD [191,192]. Anti-inflammatory dietary patterns (AIDPs) offer a promising new path in the treatment of a variety of psychiatric diseases. AIDPs like the Mediterranean diet are heavy in vegetables, fruits, and fiber, whereas pro-inflammatory Westernized diets are high in energy-dense, processed foods. AIDPs have shown high potential for modulating various mood disorders [189]. Tolkien et al. conducted an assessment of the therapeutic potential of AIDPs in depression by analyzing available scientific papers. Pro-inflammatory diets may stimulate the immune system, resulting in low-grade inflammation, and psychological disorders. Pro-inflammatory diets increase the chance of experiencing depressive symptoms or being diagnosed with depression. Because the pathophysiological underpinnings of depression and BD are so similar (e.g., depressive symptomatology, high pro-inflammatory cytokine levels), similar effects in BD are to be predicted [190]. On the other hand, a low-glycemic-index diet, which is characterized by slow-release carbohydrates and reduced insulin demand, improved mood stability, and reduced symptoms in patients with BD, possibly by regulating glucose metabolism and inflammation [191]. Additionally, a randomized controlled trial found that a dietary supplement containing omega-3 fatty acids, vitamins, and minerals improved symptoms and quality of life in patients with BD, possibly by correcting nutrient deficiencies and reducing oxidative stress [192]. Furthermore, a review of the literature suggests that a Mediterranean-style diet, which is high in fruits, vegetables, whole grains, legumes, nuts, and olive oil, may be associated with a lower risk of BD and improve mood in patients with BD, possibly due to its anti-inflammatory and antioxidant properties [193].

Diet and eating habits, as well as the contribution of nutrients that may have a good influence on BD treatment, are potential intervention goals in the treatment of BD. The data gathered are sufficient to analyze potential nutrient deficits and food habits in BD patients and to consider supplementing or dietary changes (with obvious known benefits to somatic health). Simultaneously, dietary adjustments can boost the efficacy of BD treatment by increasing the patient’s sense of control and coping. More nutritional research in BD is needed to determine the extent to which patients’ previous dietary habits, as well as the introduction of dietary interventions, may affect the pathophysiology, progression, and treatment of this illness [194,195,196,197].

## 5. Conclusions

Presented data shows that healthy dietary choices can be a non-invasive and effective strategy for combating neurologic disorders. The careful selection of dietary components can potentially affect the development and advancement of numerous neurological disorders by restoring metabolic and oxidative equilibrium and modifying inflammatory pathways in different tissues, including the brain. A diet rich in antioxidants and anti-inflammatory compounds has been shown to reduce the risk of cognitive decline in Alzheimer’s disease patients. Similarly, in Parkinson’s disease, a Mediterranean-style diet rich in fruits, vegetables, whole grains, fish, and healthy fats has been associated with a lower risk of developing the disease and a slower rate of disease progression. Additionally, certain dietary patterns such as the ketogenic diet have shown promise in managing symptoms of multiple sclerosis and Alzheimer’s disease (AD). A healthy diet that is rich in nutrients such as omega-3 fatty acids, antioxidants, and fiber can support the growth of beneficial gut bacteria and help reduce inflammation, which is a key contributor to the development and progression of neurological diseases and malaise. A diverse and healthy gut microbiome can help to promote the production of neurotransmitters, regulate inflammation, and support the integrity of the blood-brain barrier, all of which are essential for maintaining optimal neurological health. The data we collected shows that by taking care of a healthy diet and a diverse gut microbiome, individuals can actively support their neurological health and reduce the risk of developing neurological diseases. Additional research is necessary to gain a more comprehensive understanding of the intricate mechanisms by which dietary patterns and their constituents influence the etiology, advancement, and management of diverse neurological disorders. Understanding of these mechanisms could pave the way for the development of more targeted and effective dietary interventions.

## Figures and Tables

**Table 1:** The anti-inflammatory effect of diets.

Study	Study Design	Participants	Intervention	Neurological Disease	Duration	Outcome Measures	Results
Mousavi-Shirazi-Fard, Z. et al., (2021) [40]	Randomized controlled trial	100 patients with multiple sclerosis (MS)	Anti-inflammatory diet vs. usual diet	MS	12 weeks	Serum levels of inflammatory markers (CRP, IL-4, IL-17) Assessment of fatigue and quality of life (MFIS and MSQoL-54)	The anti-inflammatory diet group had significantly increased IL-4 and improvement in MFIS as well as of MSQoL-54 compared to the usual diet group
Akbari, M. et al., (2008) [41]	Randomized controlled trial	80 patients with Alzheimer’s disease (AD)	Ketogenic diet vs. usual diet	AD	12 weeks	Cognitive function (Mini-Mental State Examination), serum levels of inflammatory markers (IL-6, TNF-alpha)	The ketogenic diet group had significant improvements in cognitive function and reductions in inflammatory markers compared to the usual diet group
Phillips, M. C. L. et al., (2018) [42]	Randomized controlled trial	44 patients with Parkinson’s disease (PD)	Ketogenic diet vs. usual diet	PD	8 weeks	Serum levels of inflammatory markers motor and nonmotor symptoms	The ketogenic group showed improvements in nonmotor symptoms
Singh, B. et al., (2014) [43]	Randomized controlled trial	patients with mild cognitive impairment (MCI) or AD	Mediterranean diet vs. control diet	MCI or AD	6 months	Serum levels of inflammatory markers cognitive function	The Mediterranean diet is associated with a reduced risk of developing MCI and AD, and a reduced risk of progressing from MCI to AD.
Paknahad, Z. et al., (2020) [44]	Randomized controlled trial	80 patients with PD	Mediterranean diet vs. control diet (Iranian traditional diet)	PD	3 months	Serum levels of Total Antioxidant Capacity (TAC) and motor function (Unified Parkinson’s Disease Rating Scale)	The Mediterranean diet had a beneficial effect on TAC and on the severity of the disease
Zhang, Y. et al., (2014) [45]	Prospective cohort study	participants	Dietary patterns and risk of PD	N/A	10 years	Incident cases of PD	Higher adherence to a prudent dietary pattern was associated with a lower risk of PD
Gao, X. et al., (2007) [46]	Prospective cohort study	participants	Dietary patterns and risk of PD	N/A	16 years	Alternate Healthy Eating Index (AHEI) and the alternate Mediterranean Diet Score (aMed)	A higher AHEI or aMED score was associated with a reduced risk of PD the Western pattern increased the risk of PD
Lai, J.S., et al., (2014) [47]	Meta-analysis	participants	Dietary patterns and risk of depression	moderate to severe depression	N/A	Depression severity (BDI-II), anxiety (HADS-A), inflammatory markers	A diet high in fruits, vegetables, fish, and whole grains may be associated with reductions in depression severity and anxiety scores, as well as lower levels of inflammatory markers.
El-Mallakh, R. S. et al., (2001) [48]	Meta-analysis	patients with depression	Ketogenic diet vs. control diet	depression	N/A	Depression severity (HDRS), anxiety (HAMA), inflammatory markers (CRP, IL-6)	The ketogenic diet group had significant reductions in depression severity and anxiety scores, as well as lower levels of inflammatory markers.

## Data Availability

Not applicable.
